# Diagnosis of simulated condylar bone defects using panoramic radiography, spiral tomography and cone-beam computed tomography: A comparison study

**DOI:** 10.4317/jced.51736

**Published:** 2015-02-01

**Authors:** Fatemeh Salemi, Abbas Shokri, Hamed Mortazavi, Maryam Baharvand

**Affiliations:** 1 Assistant Professor, Department of Oral and Maxillofacial Radiology, Dental School, Hamadan University of Medical Sciences, Hamadan, Iran; 2 Associate Professor, Department of Oral Medicine, Dental School, Shahid Beheshti University of Medical Sciences, Tehran, Iran

## Abstract

Objectives: Radiographic examination is one of the most important parts of the clinical assessment routine for temporomandibular disorders. The aim of this study was to compare the diagnostic accuracy of cone-beam computed tomography(CBCT) with panoramic radiography and spiral computed tomography for the detection of the simulated mandibular condyle bone lesions. Study Design: The sample consisted of 10 TMJs from 5 dried human skulls. Simulated erosive and osteophytic lesions were created in 3 different sizes using round diamond bur and bone chips, respectively. Panoramic radiography, spiral tomography and cone-beam computed tomography were used in defect detection. Data were statistically analyzed with the Mann-Whitney test. The reliability and degrees of agreement between two observers were also determined by the mean of Cohen’s Kappa analysis. Results: CBCT had a statistically significant superiority than other studied techniques in detection of both erosive and osteophytic lesions with different sizes. There were significant differences between tomography and panoramic in correct detection of both erosive and osteophytic lesions with 1mm and 1.5 mm in size. However, there were no significant differences between Tomography and Panoramic in correct detection of both erosive and osteophytic lesions with 0.5 mm in size. Conclusions: CBCT images provide a greater diagnostic accuracy than spiral tomography and panoramic radiography in the detection of condylar bone erosions and osteophytes.

** Key words:**Bone defect, Condyle, CBCT, Panoramic, radiography.

## Introduction

Temporomandibular disorders (TMDs) are a heterogeneous group of disorders with multi-factorial etiologies. TMD is the most common cause of non-odontogenic pain in the orofacial region (1). TMDs are frequently associated with degenerative bone changes involving the bone structures of the temporomandibular joint (TMJ) such as erosion, flattening, osteophytes, subchondral bone sclerosis and pseudocysts (2). Knowledge about these bone abnormalities is fundamental for better diagnosis of dysfunctions associated with the disease and for appropriate treatment planning (3). Radiographic examination is one of the most important parts of the clinical assessment routine for TMDs. A number of imaging techniques have been developed over the last three decades; however, there is still no single method that provides accurate imaging of all the components of the complex anatomy of the TMJ (4). The TMJ structures can be viewed using panoramic and transcranial radiographs, conventional linear or complex motion tomography, computed tomography (CT), MRI and arthrography (5,6). Plain and panoramic radiographs, are good screening tools for gross bony changes. However, these tools are often limited diagnostic value because of the anatomy of the temporomandibular joint, superimpositions and the presence of overlapping structures(6,7). Although current guidelines recommend CT as the modality of choice for evaluation of TMJ osseous abnormalities, but its application in dentistry is ambient, mainly because of the cost of equipment, the large space required for its operation and the high dose of radiation involved (6-8) 

During the recent years, cone-beam computed tomography (CBCT) has been introduced in many dental institutions as a single effective method in radiographic assessment of craniofacial problems such as TMJ (1-3,9). Since the effective radiation dose with this method is still higher than that of many traditional radiographic techniques, CBCT should be substituted for such examinations only when its in case its superiority outweighs its increased positional biological radiation risk for the patient. To evaluate when CBCT is preferable in dental patients, it is prudent to compare the accuracy of CBCT for all relevant diagnostic tasks with traditionally applied methods (10), despite accreditation of CBCT for TMD diagnosis, the comparative accuracy of this method in the assessment of condylar lesions has not been addressed precisely yet (11). Therefore the aim of this study was to compare the diagnostic accuracy of CBCT imaging to conventional TMJ imaging modalities, including panoramic and spiral tomographic radiography in detection of condylar defects (erosions and osteophytes).

## Material and Methods

This was a blinded observational, in-vitro study where in sample and sample consisted of 10 TMJs from 5 dried human skulls. They were obtained from Anatomy Department, Medical Faculty, Hamadan University of Medical Sciences, Iran. No demographic data were available for the skulls. They were not identified by age, sex or ethnicity.

Condyles and temporal components of these 10 TMJs were evaluated morphologically and reported to be free from physical damage. To simulate erosive changes, a general dentist who was not among our observers created defects on the anterior-superior portion of the condyle bone with 0.5mm (small), 1mm (medium), and 1.5mm (large) in depth using a dental handpiece and a round bur with 0.5 mm indiameter (800,Tizkavan,Iran) ( Table 1). Osteophytic lesions were also simulated by using bone chips in the same sizes with the erosive ones on the anterior-superior portion of the condyle bone. We had 72 erosive lesions (0.5mm (24), 1mm (24), and 1.5mm (24)) and 72 osteophytes (0.5mm (24), 1mm (24), and 1.5mm (24)) in this study .

**Table 1:**
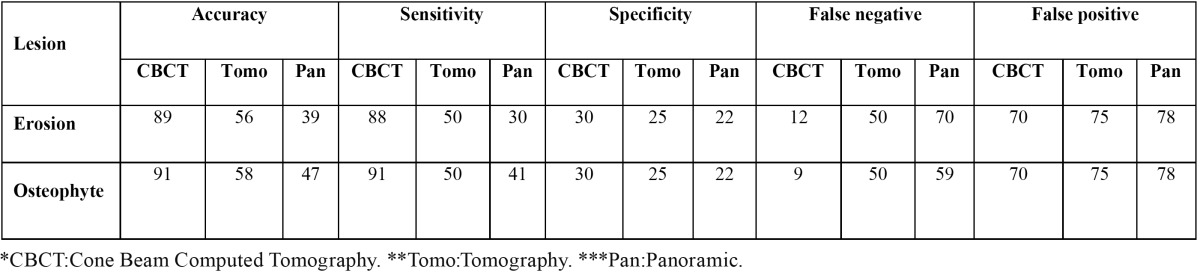
Different accuracy rate, sensitivity, specificity, false-negative, and false-positive of all studied imaging methods in detection of erosive and osteophytic lesions (%).

To provide some soft tissue attenuation, two latex balloons filled with water were placed in the cranial vault before imaging. To simulate the TMJ inter-articular space and separate the mandibular condyle from the temporal fossa a 2-mm-thick foam wedge was placed in the joint space between the glenoid fossa and the head of condyle. For all images, the remained teeth were placed in maximum inter-cuspation position and the jaws were held closed with bilateral metal springs. Skulls were also supported during the imaging by using a custom plastic head holder (11).

It is important to point out that because of our limitation in the number of intact condyles, all images (panoramic, spiral tomography, and CBCT) were taken just after creating the lesions separately. For example after creating small osteophytic lesions, images were taken, then the size of those lesions increased to moderate and imaging was done again, and this process was also repeated for large lesions. After removing osteophytic lesions from the condyl surface, erosive lesions in different sizes were created separately and images were taken by the same method, which was used for osteophytes. Images were numbered, and their characteristics were documented in a data form in terms of type and size of defects for future evaluations.

**Figure 1:**
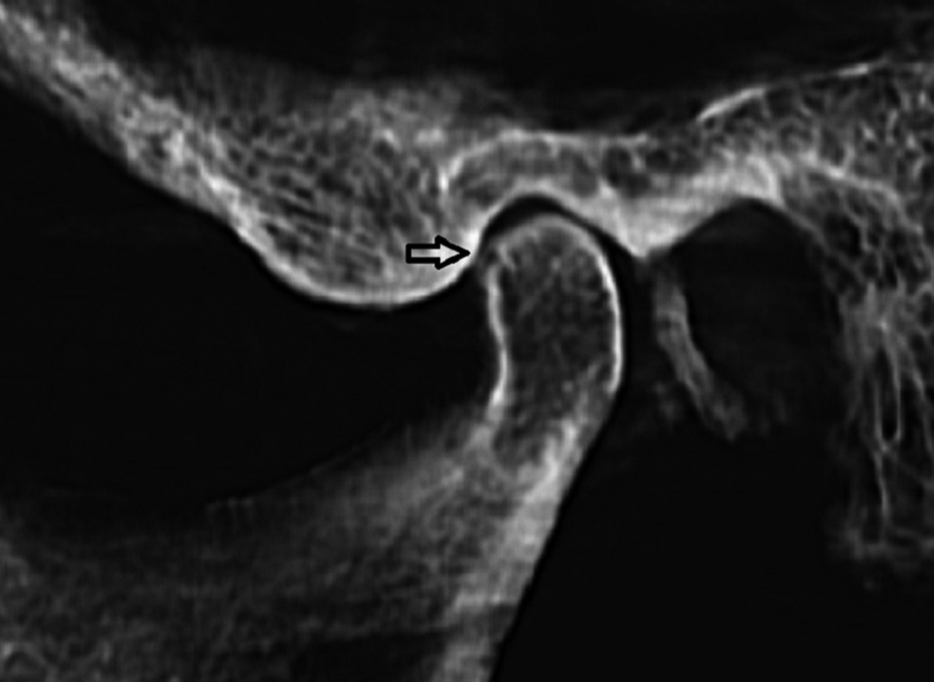
CBCT image shows sagittal view of an erosive lesion on condylar head.

**Figure 2:**
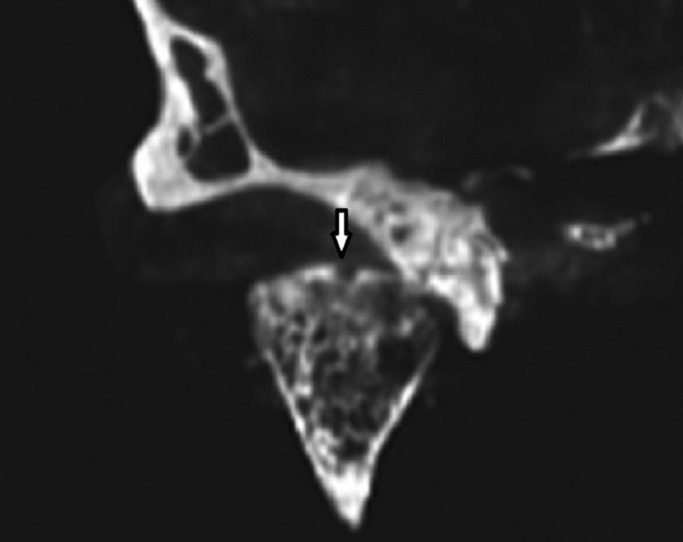
CBCT image shows coronal view of an erosive lesion on condylar head.

The CBCT images were obtained with Cranex 3D (Soredex, Helsinki, Finland), 90 kVp, 8 mA, scan time 6.1s and field of view 61×78mm (Figs.1,2). Panoramic and spiral tomography images were acquired with the Cranextom (Soredex, Helsinki, Finland) dental panoramic x-ray machine. Pan program and Lat TMJ Tomo program were selected for panoramic were 57 kVp, 6.4mA, and scan time was 15s.. Exposure parameters for panoramic were, were 57 kVp, and 1.3 mA with the scan time of 46s. These items for tomographs were 57 kVp, 1.3 mA and scan time 46s.

All images in this study were displayed on a 17-inch Samsung monitor (SyncMaster 740 N, Korea) with the screen resolution set at 1280×1024 pixels and color set to 32 bit depth. CBCT images were observed by Ondemand 3D dental software. Panoramic and tomography images were also observed using Digora software for windows.

Two calibrated blinded observers (specialist in Oral and Maxillofacial Radiology) examined images for the presence or absence of erosive and osteophytic lesions, and finally their findings compared with data form. To ensure intra-examiner reliability, the examinations were accomplished twice with at least a 6-week interval.

In this study, the diagnostic acuuracy of different techniques in detection of condylar lesions was determined according to these items: Sensitivity (true positive): Correct detection of lesion; specificity (true negative): Correct detection of a site without lesion; false positive: Detection of lesion in a site without defect; and false negative: No detection or false localization of lesion.

The reliability and degrees of agreement were also determined by the mean of Cohen’s Kappa analysis. According to the criteria of Landis and Koch, values over 0.81 was indicative of a very good or excellent agreement, 0.61 to 0.80 good or substantial agreement, 0.41 to 0.60 moderate agreement and lesser than 0.20 poor agreement (12).

Data were statistically analyzed with the Mann-Whitney test to determine differences between the imaging methods to detect simulated lesions.

## Results

In this study the values obtained for intra-examiner reliability were above 0.66 with 95% confidence interval (CI). The Kappa coefficient for inter-examiner was also 0.76 with 95% CI. The accuracy rate, sensitivity, specificity, false-negative, and false-positive of all imaging methods are summarized in  Table 1 and  Table 2 in terms of size and type of simulated lesions.

**Table 2:**
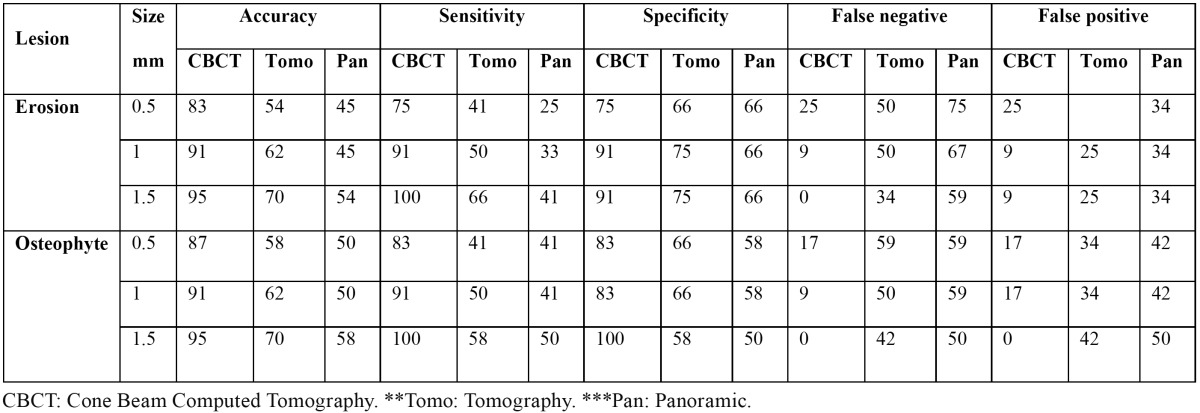
Different accuracy rate, sensitivity, specificity, false-negative, and false-positive of all studied imaging methods in detection of erosive and osteophytic lesions in terms of their sizes (%).

In this study CBCT had a statistically significant superiority over other studied techniques in detection of both erosive and osteophytic lesions with different sizes (*p*<0.05) ( Table 3). There were significant differences between tomography and panoramic view in correct detection of both erosive and osteophytic lesions with 1mm and 1.5 mm in size (*p*<0.05) ( Table 3). However, there were no significant differences between Tomography and Panoramic in correct detection of both erosive and osteophytic lesions with 0.5 mm in size (*p*>0.05) ( Table 3).

**Table 3:**
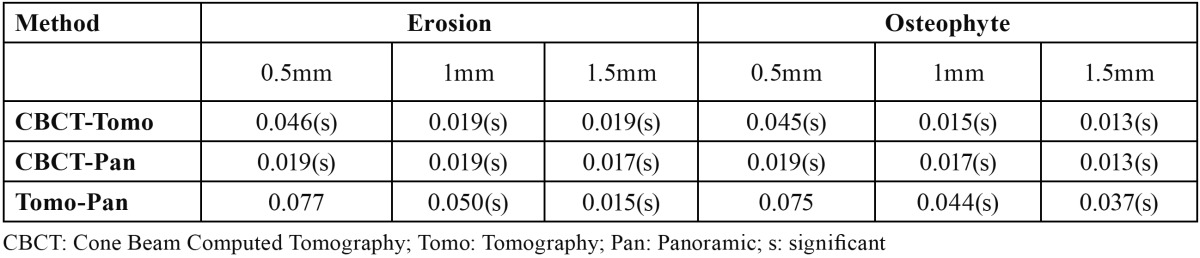
Comparison of diagnostic accuracy of different studied methods in correct detection of simulated lesions in terms of their types and sizes.

## Discussion

The aim of this study was to compare the diagnostic accuracy of CBCT with panoramic radiography and spiral computed tomography for the detection of the simulated erosive and osteophytic mandibular condyle bone defects.

According to Marques and dos Anjos Pontual, erosion and osteophyte in the mandibular condyle are prevalent in 7.9% and 3% of total TMJ alterations, respectively (2,13). Generally, erosion is the initial stage of degenerative changes, indicating that the TMJ is unstable and changes in bone surfaces will occur, probably resulting in changes in occlusion. Osteophytes occur at an advanced stage of degenerative change when the body adapts itself to repair the joint. The osteophtyes appears to stabilize and widen the surface in an attempt to improve the overload resulting from occlusal forces, representing areas of neo-formed cartilage (14). Radiologic imaging is one of the most important diagnostic tools to assess the morphology, integrity, and structural alterations of the osseous components of the TMJ (1,15). However, no single imaging method has been readily available for accurate, easily interpreted representations of all osseous aspects of the TMJ complex and associated strictures (11).

In the present study, generally, CBCT and panoramin radiography had the highest and lowest accuracy, sensitivity, and specificity in detection of both types of simulated defects. Accuracy and sensitivity of CBCT was equal in detection of osteophytes (91%). In addition, CBCT had the same specificity in detection of erosive and osteophytic defects (30%) ( Table 1).

Sensitivity (50%) and specificity (30%) of tomography were equal in both simulated lesions ( Table 1). The same result was also found for specificity (22%) of panoramic radiography ( Table 1).

Accuracy, sensitivity, and specificity of CBCT were greater than other studied methods in detection of both types of simulated defects with different sizes. CBCT had a 100% sensitivity to detect large erosive and osteophytic defects. Furthermore, specificity of CBCT was 100% for large osteophytic lesions. On the other hand, specificity of CBCT was equal in medium and large erosive lesions (91%) and small and medium osteophytic lesions (83%) ( Table 2).

A greater accuracy, sensitivity, and specificity were found for tomography than panoramic radiography in detection of both types of lesions with different sizes. Accuracy rate of tomography (54%) in detection of small erosive lesions was equal to accuracy of panoramic radiography (54%) in detection of large erosions. The same findings were also observed erosive and osteophytic defects. Sensitivity (41%) of tomography in small erosive and osteophytic lesions was equal. In addition, tomography and panoramic radiography had a similar sensitivity (41%) in detection of small osteophytic lesions. Specificity (75%) of tomography in medium and large erosions was equal. The same results were also found in small and medium osteophytes (66%) ( Table 2).

Panoramic radiography had the lowest accuracy, sensitivity, and specificity in detection of simulated lesions with different sizes in our study. Accuracy (45%) of panoramic radiography in detection of small and medium erosive lesions was equal. The same findings were reported for small and medium osteophytic lesions (50%). Small and medium osteophytic lesions and large erosive lesions were detected with similar sensitivity in panoramic radiography (41%). Specificity of panoramic radiography in all sizes of erosive lesions was 66%. Panoramic radiography also showed a similar specificity (58%) in detection of small and medium oste-ophytic lesions ( Table 2).

Our study demonstrated that CBCT had a statistically significant superiority over other studied techniques in detection of both erosive and osteophytic lesions with different sizes.

In addition, we found that there were statistically significant differences between Tomography and Panoramic radiography in correct detection of medium and large erosive and osteophytic lesions. However, there were no statistically significant differences between Tomography and Panoramic radiography in correct detection of small erosive and osteophytic lesions ( Table 3).

In agreement with our study, Honey demonstrated that CBCT provides superior reliability and greater accuracy than panoramic radiography and tomography in detection of condylar erosions (11). Tsiklakis, Honda, Hilgers, Wang and Zhang have reported a high dimensional accuracy of CBCT in detection of condylar defects (16-20).

CBCT also provides multiplanar images in the anatomic sagittal, coronal and axial planes. For easier TMJ visualization, the image volume can be reconstructed in planes parallel and perpendicular to the long axis of the condyle instead of true anatomic coronal and sagittal planes. These reconstructed sections allow better evaluation of the condyle within the glenoid fossa (1).

In contrast to our findings, Hintze compared CBCT with conventional tomography in detection of TMJ bone pathologies and found that there were no significant differences in diagnostic accuracy between these two techniques (10). In addition, Patel evaluated CBCT in the diagnosis of simulated small osseous defects in the mandibular condyle and found that detection of lesions smaller than 2mm can be difficult (21).

Tomography has been used for the second sensitivity and accuracy rate in diagnosis of simulated condylar bone defects after CBCT in our study. Tomography has been used for several years as the modality of choice for TMJ bone components. According to Flygare and Cholitgul, sensitivity of tomography for detection of osseous changes ranges from 53% to 90% and the specificity ranges from 73% to 95%, which are close to our results (22,23).

Our results showed that panoramic radiography had the lowest accuracy and sensitivity for detecting simulated lesions in the condylar bone. This finding is in agreement with Ahmed and Crow (24,25). Although Panoramic radiography has been suggested for evaluation of TMJ osseous abnormalities, there are several limitations that minimize its value for TMJ assessment. This technique does not show the entire articular surface of the TMJ. Meanwhile, radiographs are distorted, and often there is superimposition from zygomatic process (1).

## Conclusions

CBCT images provide a greater diagnostic accuracy than spiral tomography and panoramic radiography in the detection of condylar bone erosions and osteophytes.
